# Editorial: The role of transcriptional regulation in mycobacterium physiology

**DOI:** 10.3389/fcimb.2024.1483263

**Published:** 2024-09-02

**Authors:** Siguo Liu, Ningning Song, Selvakumar Subbian

**Affiliations:** ^1^ Harbin Veterinary Research Institute, Chinese Academy of Agricultural Sciences, Harbin, China; ^2^ Key Laboratory of Respiratory Tract Pathogens and Drug Therapy, School of Life Science and Technology, Shandong Second Medical University, Weifang, China; ^3^ Public Health Research Institute, New Jersey Medical School, Rutgers University, Newark, NJ, United States

**Keywords:** host response, transcription regulation, BCG, omics, non-tuberculous mycobacteria

## Introduction

*Mycobacterium tuberculosis* (Mtb) is an intracellular pathogenic bacterium that can cause a spectrum of clinical outcomes, ranging from a highly contagious and potentially lethal tuberculosis (TB) to asymptomatic latent infection (LTBI) in humans and animals (Klever et al., 2023). In addition, the asymptomatic LTBI cases can reactive to symptomatic TB upon immune-suppressing host conditions. Thus, the morbidity and mortality due to TB with or without co-morbid conditions are significant health issues worldwide. Upon infection with Mtb, the host cells produce several cytokines and chemokines that facilitates the recruitment and activation of immune cells from the blood to the site of infection, resulting in the formation of granuloma. These highly cellular structures can undergo “maturation” with time to form necrotic centers that further can develop into cavities (Ashenafi and Brighenti, 2022). To survive within the necrotic granuloma, the infecting Mtb must adapt to the hostile microenvironment, which is characterized by hypoxia and nutrient scarcity (Orgeur et al., 2024). Although the current antibiotic regimen, comprised of rifampicin (RIF), isoniazid (INH), pyrazinamide (PZA) and ethambutol (ETH) has been widely successful in curing active TB, the treatment outcome may be worsened by the presence of drug resistant (DR) strains of Mtb. The appearance of multi-drug resistant (MDR) and extremely drug-resistant (XDR) Mtb strains can reduce the treatment success in TB and contribute to the transmission of MDR and XDR TB cases, which are extremely difficult to cure (Gupta et al., 2024; Jones et al., 2024).

One of the difficulties associated with the eradication of TB through antibiotic treatment is the ability of Mtb to persist in a dormant state within the infected host and maintain viability in the absence of replication. The adaptation of microbial pathogens to the host environment requires integration of a myriad of signaling pathways as well as tight control of both function and dynamics of regulatory networks. Mtb has about 200 transcriptional regulatory proteins, which play key roles in the persistence, drug resistance, pathogenesis, amino acids biosynthesis of the bacteria. The concerted effect of these functions is essential for the successful survival of Mtb in the changing host environment (Orgeur et al., 2024). Therefore, deciphering the regulatory mechanism of Mtb to adapt to the host environment using various analytical methods, including transcriptomics, proteomics and metabolomics under different growth environments of the bacteria, is vital to gain knowledge on our understanding of TB pathogenesis.

The Research Topic entitled “The Role of Transcriptional Regulation in Mycobacterium Physiology” presents a series of articles that highlight the advanced studies for regulation in Mycobacterium. The Research Topic is comprised of four peer-reviewed manuscripts in the fields of molecular biology, microbiology and bioinformatics related to mycobacterial physiology and pathogenesis. This Research Topic is focused on deciphering the mechanism of regulation and host-pathogen interactions of mycobacteria, which would help in the discovery of potential and novel drug targets and the development of new anti-TB drugs.

In the original research article of Cui et al., the authors presented data from experiments that decipher the novel regulatory function of DosR (dormancy survival regulator; Rv3133c), which is one of the key transcriptional proteins regulating bacterial dormancy and participating in various metabolic processes (Liu et al., 2024). This combined omics analysis finds that deleting DosR significantly affects the transcriptional levels of 104 genes and 179 proteins in *M. bovis* BCG, an attenuated strain of the virulent pathogen *M. bovis*. Targeted metabolomics data for amino acids indicate that DosR knockout strain significantly upregulates L-Aspartic acid and L-Serine synthesis, while downregulating seven other amino acids, including L-Histidine and L-Lysine. Additionally, compared to the wild-type (WT) strain, the DosR-knockout strain produced a higher level of reactive oxygen species (ROS), and a significant increase in DNA damage. These observations indicate the role of DosR on the antimicrobial responses of mycobacteria. Furthermore, the electrophoretic mobility shift assay (EMSA) demonstrated that DosR may regulate many targeted genes that have not been explored previously. In summary, this study further expands the regulatory role of DosR on bacterial adaptation to stress conditions and indicate that DosR may be a potential candidate target for TB drug development.

In addition to Mtb, other pathogenic and non-pathogenic mycobacterial species have been reported to adapt effectively through two-component (TC) signal transduction systems to survive in various environmental growth niches (Stupar et al., 2022). The article by Płocinska et al. describes the role of a novel TC system, comprised of a sensor kinase, N,N-dimethyl-p-nitrosoaniline (NDMA)-dependent methanol dehydrogenase (Mno)-S and a response regulator, MnoR, on the regulation of methylotrophic and other metabolic processes in *M. smegmatis*. Using deletion mutants and complementing strains of the MnoSR operon system, the authors performed RNA-seq-based genomewide transcriptome analysis of *M. smegmatis* under different nutritional conditions. Results from further molecular analysis indicate that MnoSR play an important role in the adaptation of *M. smegmatis* to use alcohols, including 1,3-propanediol and ethanol and glucose as carbon sources. In summary, this study highlights the MnoSR system’s vital role on the metabolic adaptability of mycobacteria to survive in different nutritional conditions.

During pulmonary infection, pathogenic mycobacteria are primarily engulfed by the alveolar macrophages; however, the molecular responses of these infected host cells are not fully understood (Ghoshal et al., 2024). In the article by Cai et al., a comparative proteomic analysis of bovine alveolar macrophages (BAMs) conducted after infection Mtb or *Mycobacterium bovis* (Mbo) to elucidate the differential responses of BAMs to these pathogens. Results from this study show that BAMs may resist Mtb and Mbo by different mechanisms. Thus, Mtb and Mbo infection triggered divergent but significant changes in the energy metabolism of infected BAMs. Similarly, several proteins and signaling pathways associated with autophagy and inflammation were upregulated in Mbo-infected BAMs, compared to Mtb infection. Overall, this study provides critical insights into the response of BAMs to Mtb and Mbo infections, unveiling potential targets to facilitate more effective treatment strategies.

Recent clinical studies indicate that non-tuberculous mycobacteria (NTM) can cause significant morbidity, particularly in immunocompromised individuals (Gopalaswamy et al., 2020). Similar to Mtb, NTM species such as *Mycobacterium abscessus* (Mab) can infect and survive within macrophages, by resisting the antimicrobial effects of these host cells (Gopalaswamy et al., 2020; Gopalaswamy et al., 2021). However, the intracellular adaptation mechanisms utilized by NTM species are not fully understood. The research article by Simcox et al. investigates the role of *nnaR* (nitrate/nitrite assimilation regulator), an orphan response regulator, on the adaptation of Mab to assimilate nitrate and nitrite. The authors determined the domain structure and sequence alignment of NnaR using bioinformatics analysis and explored the genomic and transcriptional organization of Mab NnaR regulon. Using *nnaR* knockout (*Mab_ΔnnaR_*) and complementing (*Mab_ΔnnaR+C_*) strains of Mab, the authors tested the role of NnaR in nitrate and nitrite utilization by Mab. Results show that *nnaR* regulates the *narK3*-*nirBD*-*nnaR*-*sirB* operon and *nasN*, all of which are crucial for nitrogen metabolism in Mab, and the Mab*_ΔnnaR_* strain was unable to positively regulate these nitrogen utilization genes, and to grow in nitrate-containing growth medium. Thus, the authors have established that NnaR is required for nitrogen metabolism in Mab. This study highlights one of the survival mechanisms used by Mab to persist in the host. Future studies on the exploration of signaling pathways that regulate NnaR activation would aid in a better understanding of Mab persistence in various host immune cells and help to identify therapeutic targets to control Mab infections.

In summary, this Research Topic includes articles on the molecular determinants of mycobacterial adaptation strategies to survive under various selection pressure, and the host cell responses to infection by mycobacteria. The findings presented in these articles advance our knowledge on the pathogenesis of mycobacterial infections. In addition, the molecules and/or pathways reported in these studies may be extrapolated to understand the pathogenesis of other infectious agents. Together, these discoveries would provide the knowledge and platform for devising better and efficient intervention measures, such as host-directed therapies for TB and other infectious diseases (Kolloli and Subbian, 2017).
